# Non-ST-Elevation Myocardial Infarction as the Initial Manifestation of Calreticulin-Positive Essential Thrombocythemia: A Case Report

**DOI:** 10.3390/jcdd12040157

**Published:** 2025-04-16

**Authors:** Jared B. Hinton, Jalal S. Jwayyed, Sonum Jagetia, Hunter J. Landwehr, John D. Scrocco

**Affiliations:** 1 Department of Medicine, Northeast Ohio Medical University, Rootstown, OH 44272, USA; jjwayyed1@neomed.edu (J.S.J.); sjagetia@neomed.edu (S.J.); hlandwehr@neomed.edu (H.J.L.); 2 Department of Cardiology, Mercy Health St. Elizabeth Boardman Hospital, Boardman, OH 44512, USA; jdscroc@neomed.edu

**Keywords:** essential thrombocythemia (ET), non-ST-elevation myocardial infarction (NSTEMI), CALR mutation, myeloproliferative neoplasm (MPN), thrombocytosis, acute coronary syndrome (ACS), thrombotic complications

## Abstract

Essential thrombocythemia (ET) is a rare myeloproliferative neoplasm characterized by excessive platelet production and a predisposition to thrombotic or hemorrhagic complications. We report a case of a 62-year-old male with no conventional cardiovascular risk factors who presented with a non-ST-segment elevation myocardial infarction (NSTEMI). Initial coronary angiography showed isolated proximal LAD stenosis. Laboratory tests revealed marked thrombocytosis (>1,000,000/μL) and a CALR mutation, confirming a diagnosis of ET. The patient was treated with percutaneous coronary intervention (PCI), dual antiplatelet therapy, and cytoreductive therapy with hydroxyurea, leading to a favorable outcome. This case illustrates how ET, particularly CALR-mutated subtypes, can manifest as acute coronary syndrome in the absence of atherosclerosis and underscores the need to consider hematologic malignancies in atypical presentations of myocardial infarction.

## 1. Introduction

Myocardial infarction (MI) is a major global health concern, affecting nearly 3 million people worldwide and accounting for over 1 million deaths annually in the United States alone [1]. The classic pathophysiology of MI involves the rupture of an atherosclerotic plaque, followed by platelet activation, aggregation, and thrombus formation [2]. However, in the absence of significant atherosclerotic disease, clinicians must consider less common etiologies such as coronary artery spasm, embolism, or underlying hematologic disorders.

Essential thrombocythemia (ET), a chronic myeloproliferative neoplasm (MPN), is characterized by clonal proliferation of megakaryocytes, persistent thrombocytosis, and an elevated risk of both arterial and venous thrombosis [3]. Among hypercoagulable states, ET is a rare but clinically significant contributor to arterial thrombotic events, including MI. Platelet hyperreactivity and elevated counts, hallmarks of ET, can significantly enhance thrombus formation, particularly in the context of endothelial injury or systemic inflammation [4].

Approximately 90% of ET cases are associated with somatic mutations in the JAK2, CALR, or MPL genes, with CALR mutations accounting for about 23% [5]. Although CALR-positive ET is typically associated with a lower thrombotic risk than JAK2-positive cases, thrombotic complications can still occur, especially in the context of extreme thrombocytosis, older age, or other modifying factors. Arterial thromboses, including myocardial infarction (MI), have been reported in up to 14% of patients at diagnosis and remain a major source of morbidity in this population [5].

In parallel, cardiovascular complications have emerged as increasingly recognized adverse effects of modern cancer immunotherapies. Immune checkpoint inhibitors (ICIs), chimeric antigen receptor (CAR)-T cell therapies, cancer vaccines, and other immune-modulating agents have revolutionized cancer care but are associated with immune-related adverse events (irAEs) that include myocarditis, pericarditis, and arterial thromboembolic events [6]. As highlighted by Panuccio et al. (2025), these immune-mediated cardiovascular toxicities often mimic classic acute coronary syndromes and present diagnostic challenges, particularly in patients with concurrent or underlying hematologic abnormalities [6]. Despite existing cardio-oncology guidelines, a tailored approach is necessary to manage these overlapping syndromes effectively, especially in patients at risk for both malignancy- and therapy-driven vascular events.

Although our patient was not actively undergoing immunotherapy or cancer treatment, his presentation illustrates the overlapping clinical and pathophysiologic features between MPN-associated thrombosis and immunotherapy-related cardiovascular syndromes. Both conditions can be driven by inflammation, endothelial activation, immune dysregulation, and thrombosis. This overlap reinforces the importance of maintaining a broad differential diagnosis when evaluating acute coronary syndromes in atypical patients, including those with thrombocytosis or no conventional cardiovascular risk factors.

We present a case of a 62-year-old male with CALR-mutated ET who developed a non-ST-elevation myocardial infarction (NSTEMI) as the initial manifestation of his disease. The case underscores the critical need to consider MPNs such as ET in patients presenting with ACS in the absence of significant atherosclerotic disease, and it reflects the broader implications of cardiovascular complications in hematologic and oncologic disorders. This report highlights the diagnostic challenge and clinical relevance of such atypical presentations.

## 2. Case Presentation

### 2.1. Patient Presentation

A 62-year-old male, lifelong nonsmoker with no prior history of hypertension, hyperlipidemia, diabetes, or coronary artery disease, presented to the emergency department with a 6-week history of episodic midsternal “burning” pain. The pain, occasionally radiating to his back, was associated with dysphagia, abdominal bloating, and palpitations. The symptoms were initially attributed to gastroesophageal reflux disease but later became persistent and unresponsive to antacids. On the day of admission, the patient experienced prolonged chest discomfort that resolved before arrival at the emergency department.

### 2.2. Initial Evaluation

**Figure 1:**
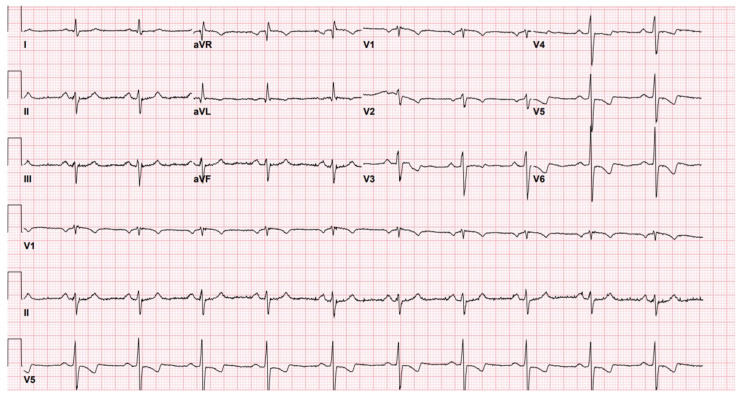
EKG findings showing lateral ST and T-wave abnormalities.

Upon arrival, the patient’s vital signs were stable, with a blood pressure of 138/89 mmHg, a heart rate of 68 bpm, and an oxygen saturation of 98%. An electrocardiogram (EKG) revealed an NSTEMI with a sinus rhythm and lateral ST and T-wave abnormalities (Figure 1). These findings prompted further evaluation, but physical examination revealed no significant abnormalities, with clear lung fields, no murmurs, and no peripheral edema. Serial high-sensitivity cardiac troponin T (hs-cTnT) levels were elevated, rising from 84 ng/L to a peak of 123 ng/L before trending down to 104 ng/L, confirming myocardial injury consistent with non-ST-elevation myocardial infarction.

### 2.3. Diagnostic Workup

Further diagnostic testing included routine blood tests and imaging. Figure 1 displays the EKG findings illustrating lateral ST and T-wave abnormalities. A chest X-ray showed no acute abnormalities, and an echocardiogram revealed preserved left ventricular function with an ejection fraction of 60% and no regional wall motion abnormalities. Cardiac catheterization demonstrated an 80% proximal LAD stenosis with TIMI-3 flow and no significant disease in other coronary vessels.

Post-procedurally, the patient was admitted to the cardiac intensive care unit for monitoring and further evaluation. Given the atypical presentation—acute MI in the absence of atherosclerotic disease—an extensive workup was initiated to identify potential underlying causes of the thrombotic event. Initial laboratory investigations revealed a profoundly elevated platelet count, exceeding 1,000,000/μL. Routine coagulation studies, including prothrombin time (PT), activated partial thromboplastin time (aPTT), and fibrinogen levels, were all within normal limits. Additional inflammatory markers, such as C-reactive protein (CRP) and erythrocyte sedimentation rate (ESR), were mildly elevated but lacked specificity. These findings prompted hematologic evaluation and molecular testing. Molecular testing identified a Calreticulin (CALR) mutation (a 52 bp deletion), while JAK2 and MPL mutations were negative, confirming a diagnosis of ET [7].

### 2.4. Management

The patient was initially managed with intravenous heparin, high-dose atorvastatin, and dual antiplatelet therapy (aspirin and ticagrelor, later transitioned to clopidogrel). Coronary perfusion was restored via aspiration thrombectomy. Percutaneous coronary intervention (PCI) was performed for an 80% ostial proximal LAD stenosis with TIMI-3 distal flow. Using a 6 French EBU 3.5 guide catheter and a Runthrough wire, a 3.0 × 15 mm Euphora balloon was inflated multiple times at nominal pressure, followed by deployment of a 4.5 × 22 mm Frontier Onyx stent at 12 atm. The final angiography showed 0% residual stenosis and maintained TIMI-3 flow. Intravascular ultrasound confirmed minimal plaque beyond the stent and no edge dissection. Cytoreductive therapy with hydroxyurea was initiated to address thrombocytosis and mitigate the risk of recurrent thrombotic events. Risk factor modification, including lifestyle counseling and close follow-up, was emphasized throughout the patient’s care.

### 2.5. Outcomes

Post-PCI, the patient was monitored in the cardiac intensive care unit, where platelet counts gradually normalized with hydroxyurea therapy. Follow-up echocardiography demonstrated preserved cardiac function, and the patient reported complete resolution of symptoms. The patient was educated on recognizing symptoms of thrombosis or bleeding and the potential complications of ET, such as transformation to myelofibrosis or progression to acute myeloid leukemia, although these risks remain low in well-managed cases. He was discharged with recommendations for regular hematologic and cardiologic follow-up, and his recovery was uneventful.

## 3. Discussion

The presentation of acute coronary syndrome (ACS) in patients with essential thrombocythemia (ET) is rare but critical to recognize, as delayed diagnosis can lead to recurrent thrombotic events and increased morbidity. In this case, the absence of significant atherosclerosis and traditional cardiovascular risk factors showed the importance of considering alternative etiologies, such as ET, in cases of unexplained thrombosis [7]. The discovery of markedly elevated platelet counts and a CALR mutation confirmed the diagnosis of ET, which likely contributed to the thrombotic event.

One unique feature of this case is the presentation of NSTEMI as the initial clinical manifestation of ET. While arterial thrombosis is a recognized complication of ET, CALR-mutated ET is typically associated with a lower thrombotic risk compared to JAK2-mutated disease [8]. However, this patient’s extreme thrombocytosis (>1,000,000/μL) likely played a central role in creating a prothrombotic milieu. In ET, thrombosis is not solely driven by platelet count but also by qualitative platelet abnormalities, inflammatory cytokines, aberrant megakaryocyte activity, and endothelial dysfunction. In CALR-mutant ET specifically, emerging evidence suggests that despite a lower overall thrombotic risk, mutant CALR can activate the thrombopoietin receptor (MPL) and promote megakaryopoiesis and platelet hyperreactivity [8,9]. This underscores the multifactorial nature of thrombosis in ET and the need for a high index of suspicion when patients present with acute coronary syndromes and unexplained thrombocytosis. CALR-positive ET, as per the 2016 WHO classification, represents a distinct molecular subset of ET with a generally lower thrombotic risk profile [10], yet this case illustrates that such patients may still present with arterial thrombosis in atypical ways.

This case also highlights a broader challenge in clinical cardiology: the diagnostic ambiguity in patients presenting with MI in the absence of obstructive coronary artery disease (MINOCA). In such cases, clinicians must expand the differential to include hypercoagulable disorders, vasospasm, myocarditis, and hematologic malignancies. Elevated platelet counts should prompt an early consultation with a hematologist, as delays in diagnosis may lead to recurrent thrombotic episodes [3].

Most patients with ET present with nonspecific symptoms such as fatigue, microvascular disturbances or are diagnosed incidentally after routine blood work reveals thrombocytosis [9]. Arterial thrombotic events, when they do occur, are more common in patients with JAK2 mutations and often affect individuals with traditional cardiovascular risk factors. In contrast, this patient had CALR-mutated ET, no classic cardiovascular comorbidities, and presented with an NSTEMI as the initial manifestation, making the case atypical compared to the broader ET population. He had no conventional cardiovascular risk factors such as hypertension, diabetes, dyslipidemia, smoking, or a history of coronary artery disease. Coronary angiography confirmed the absence of significant atherosclerosis. Given his marked thrombocytosis and CALR-driven platelet dysfunction, the thrombotic event was most likely driven by the prothrombotic and inflammatory milieu associated with ET, despite the absence of other identifiable triggers.

Interestingly, while our patient was not undergoing active cancer treatment, his presentation mirrors emerging patterns seen in cardio-oncology, where immune-mediated mechanisms—including inflammation, endothelial injury, and thrombosis—underlie cardiovascular events in cancer patients receiving immune checkpoint inhibitors, CAR-T therapies, or cancer vaccines [6]. As highlighted by Panuccio et al., the overlap between hematologic malignancies and cardiovascular complications is increasingly evident, particularly in cases where immune dysregulation drives vascular injury. This reinforces the importance of considering hematologic or therapy-related etiologies in patients with acute coronary syndromes and no overt cardiovascular risk factors, especially in the era of expanding cancer therapeutics.

Patients with ET can present with a broad spectrum of clinical manifestations beyond coronary artery thrombosis. Priapism is a rare potential presentation of ET, which can have severe consequences, including irreversible erectile dysfunction if not promptly treated [11]. Additional presentations include venous thrombosis, microcirculatory disturbances such as erythromelalgia, and complications like digital ischemia and transient ischemic attacks [11]. Venous thrombosis often involves unusual sites, such as the splanchnic veins, and may be the first indication of an underlying myeloproliferative disorder. Recognizing these atypical presentations is critical for timely diagnosis and intervention.

The management of ET is tailored to the level of risk, with a focus on preventing thrombotic complications. High-risk patients with ET are defined by age over 60 years, prior thrombotic events, or the presence of JAK2 mutations and benefit most from cytoreductive therapy [9]. Hydroxyurea remains the first-line treatment for reducing platelet counts and preventing future thrombotic events. Second-line treatments like interferon-α or busulfan may be considered in cases of intolerance or resistance to hydroxyurea [10]. Unless contraindicated, aspirin therapy is recommended for all patients with ET, particularly for those with JAK2 mutations and cardiovascular risk factors.

In cases of coronary thrombosis, as in this patient, revascularization with PCI is the cornerstone of management. Early initiation of cytoreductive therapy before PCI may reduce the risk of the no-reflow phenomenon caused by platelet aggregation and activation [6]. In this case, the combination of cytoreductive therapy and antiplatelet agents was effective in preventing early recurrent thrombosis.

Patients with CALR-mutated ET often present with unique clinical profiles, with a generally lower thrombotic risk than JAK2-positive cases [12]. However, arterial thrombosis, including coronary events, can still occur and warrants careful monitoring and management. This demonstrates the evolving understanding of molecularly stratified ET and its implications for personalized treatment approaches.

The implications for clinical practice are significant: routine blood counts and mutation screening in atypical MI presentations may uncover underlying myeloproliferative neoplasms, allowing for the earlier initiation of disease-modifying therapies such as hydroxyurea and more tailored antithrombotic regimens. Previous case reports have noted myocardial infarction as the initial manifestation of ET, yet CALR-mutated cases remain rarely described [13].

## 4. Conclusions

This case highlights the critical importance of considering ET in the differential diagnosis of ACS, particularly in patients presenting with thrombocytosis and no traditional cardiovascular risk factors. Early recognition, molecular diagnostics, and a multidisciplinary approach remain essential for optimizing outcomes and mitigating complications. This report shows the variability of ET presentations, ranging from arterial thrombosis to venous thromboembolism, priapism, and erythromelalgia, emphasizing the need for clinical vigilance in atypical cases.

Furthermore, this case demonstrates the effectiveness of integrating personalized treatment strategies, such as cytoreductive therapy, aspirin, and PCI, in managing ET-related thrombotic events. It contributes to the growing body of evidence on CALR-mutated ET, underscoring its distinct clinical profile and thrombotic potential, even in patients at lower risk. Future research should focus on refining risk stratification tools, evaluating the prognostic implications of molecular mutations, and exploring the long-term outcomes of novel therapies such as pegylated interferons and direct oral anticoagulants. A tailored approach will remain pivotal in advancing care and improving outcomes for patients with ET.

## Data Availability

No new data were created or analyzed in this study. Data sharing is not applicable to this article.

## Abbreviations

The following abbreviations are used in this manuscript:

ET	Essential Thrombocythemia
NSTEMI	Non-ST-Elevation Myocardial Infarction
MPN	Myeloproliferative Neoplasm
ACS	Acute Coronary Syndrome
PCI	Percutaneous Coronary Intervention
EKG	Electrocardiogram
LAD	Left Anterior Descending (Artery)
TIMI	Thrombolysis in Myocardial Infarction
PT	Prothrombin Time
aPTT	Activated Partial Thromboplastin Time
CRP	C-Reactive Protein
ESR	Erythrocyte Sedimentation Rate
AML	Acute Myeloid Leukemia
VTE	Venous Thromboembolism
